# On-chip Microscopy Using Random Phase Mask Scheme

**DOI:** 10.1038/s41598-017-14517-3

**Published:** 2017-11-07

**Authors:** Anwar Hussain, Yicheng Li, Diyi Liu, Cuifang Kuang, Xu Liu

**Affiliations:** 1 State Key Laboratory of Modern Optical Instrumentation, College of Optical Science and Engineering, Zhejiang University, Hangzhou, 310027 China; 2 Collaborative Innovation Center of Extreme Optics, Shanxi University, Taiyuan, 030006 China; 3 Quantum Optics Lab, Department of Physics, COMSATS Institute of Information Technology Islamabad, Islamabad, Pakistan

## Abstract

In this study, a simple and novel phase-retrieval scheme is implemented using multi-angle illumination to enhance the resolution of lensless microscopy. A random-phase mask (from 0 to 2π) precedes the sample to encode the information at the sensor plane. The sample is illuminated with multiple angles that are symmetrical along the optical axis of the system. The system is initially calibrated while recording the images without any sample at the corresponding multi angles. The two types of image are mutually subtracted, and the resultant images are summed at the sensor plane and backpropagated to the sample plane. The final image is free of the twin-image effect, and has a high signal-to-noise ratio owing to the multi angles of the illumination scheme. This scheme gives a resolution of ~4 micron for a large field-of-view (~15 mm^2^). The scheme is useful for robust imaging owing to the fast phase-retrieval method, and it enables a straightforward analytical reconstruction instead of using complicated iterative algorithms in a lensless microscopic setup.

## Introduction

Microscopic imaging techniques are usually categorized into confocal and wide-field classes based on the field-of-view (FOV). The choice of class depends upon whether the researcher requires a large FOV or point-to-point imaging. The earlier class of systems uses objective lenses to collect and magnify the sample information at the sensor plane^1–3^. However, the FOV of such optical systems is restrictive owing to its high objective lens magnification. These systems are used for coherent imaging^4^, confocal imaging^5^, and stimulated emission depletion (STED)^6^ imaging. The numerical aperture of an objective lens allows only low spatial frequencies to pass, while higher spatial frequencies of the sample are blocked and the resultant image is blurred. As a result of imaging lens imperfections, the aberration effects are also added into the imaging system. The removal of the imaging lens from the optical system, i.e., using lensless imaging, not only avoids these drawbacks but also introduces a few additional positive features to the imaging system. The fundamental positive feature of lensless microscopy is that the FOV is independent of the magnification (unit in this case). The second feature is that it transforms the geometrical shape of an optical system into a more compact, portable, and less expensive one. In lensless microscopy, the sample is placed very close to the sensor and illuminated with a perpendicular light source from an optimum height. The diffracted light reaching the sensor plane appears either as a blurred spot for an incoherent source or as interference fringes for a coherent source. The recorded image is reconstructed in the sample plane with low resolution owing to the large pixel size and missing higher spatial frequencies. Lensless imaging is also used as bio-samples to monitor the cell division, viability, and motility of cells^7–9^ with high precision. Lensless microscopy can be divided into two main processes, i.e., raw imaging of the sample and the computational retrieval of sample information. The first step is the experimental capturing of images at the sensor plane, and the second one is the reconstruction of a resultant image with enhanced resolution and improved signal-to-noise (SNR) ratio. The reconstruction of images from the recorded in-line holograms at the sensor is performed digitally, and results from scattered light due to the features of the sample and un-scattered light passing through the transparent part of the sample. During the reconstruction process, the image is transformed to the spectrum and multiplied using the free-space propagation transfer function^10,11^. In the sample plane, the inverse Fourier transform of the image is obtained to obtain the optical field of the sample. This simple reconstruction process encountered several limitations, which are further compensated through alternate approaches to yield high-resolution images. Then, the recorded images are propagated back and forth between the different planes to retrieve the lost phase of a sample. The second limiting factor of the reconstruction process is the size of the sensor pixel. To minimize the negative effect of image pixilation, the size of each pixel is reduced to the minimum possible size, which corresponds to an increase in the number of pixels for the same sensor active area. The sample disturbance having spatial frequencies smaller than 2/λ will reach the sensor, and the remaining ones will decay before reaching the sensor plane as the separation between the sensor and sample is greater than a few wavelengths. The pixel size (~1 µm) is greater than λ/2, which leads to a resolution gap at the sensor plane. To resolve this resolution gap, the pixel super-resolution technique was implemented^12,13^. In this technique, one of the optical components (sensor or source of the illumination or sample) is shifted in the x-y plane with sub-pixel steps. The shifting of the source corresponds to a shift in the hologram in the sensor plane, and a large number of raw images are recorded with a virtually smaller pixel size than the physical sensor pixel. The summation of these images results in a final image with smaller pixel size compared to the actual pixel, and this small pixel is equal to or less than the value of λ/2. The pixel super-resolution method has been combined with the synthetic aperture method, where the sample is illuminated from different angles to shift the higher spatial frequencies to low spatial frequencies^10,14^. Using this technique, the achieved spatial resolution of ≤1 µm is decreased to 250 nm, and the overall numerical aperture of the system is also synthesized to a value of 1.4. Then, the pixel super-resolution was combined with wavelength scanning^15^, where the sample is illuminated with different wavelengths at different heights to record raw images. To minimize the effect of twin images in lensless microscopy, the optical component, which is called a phase mask, has been introduced to encode the sample information at the sensor plane^16,17^. The image retrieved with the phase-masking technique has a reduced twin-image effect with a low SNR.

In this study, the simple phase-mask technique was implemented for phase retrieval. The random-phase mask was used before the sample plane to encode the sample information during propagation to the sensor plane. The phase mask is illuminated with plane waves having a propagation direction parallel to the optical axis of the system. The recorded image at the sensor carries the sample information as well as the plane-wave and phase-mask information. The main challenge after capturing an image is the successful retrieval of real image of the sample. The extra phases carried by the recorded image introduce aliasing and speckle noise, which are evenly distributed throughout the image. The other type of image, which is captured by calibration of the system, is subtracted from the sample image. The net resultant image is then backpropagated to the sample plane using the angular spectrum propagation method in the negative z-direction. The image contains the information of the phase mask, which appears as speckle noise in the propagated image. To obtain the final image, the image without the sample is backpropagated to the sample plane, and the original image is divided using this propagated pattern. This process decreases the aliasing effect, and the twin image is also suppressed in the phase pattern. However, the SNR of the retrieved image is low owing to the wave nature of light. To further increase the resolution of the retrieved image, the multi-angle illumination technique was implemented. The sample is scanned through the plane wave while changing the angle of illumination along the optical axis from −30° to +30° in order to introduce the higher spatial frequencies into the pass band. 31 images are captured in this process. The sample is then in-plane rotated for 90° with respect to the initial system coordinates, and the previous procedure is repeated to capture another 31 images. A total of 93 images were recorded, including 62 images with the sample and 31 images without the sample. In our case, the sample is scanned along the x- and y-axes as the sample USAF 1951 resolution chart consisted elements along these directions. The images retrieved along both directions are then summed to obtain the final image. The scheme was tested using computer simulations and by performing experiments. Both testing processes validate our theoretical model and new applications in lensless microscopy are proposed.

### Theory of phase-masking scheme

The mathematical model is presented to explain the idea of the multi-angle illumination scheme in lensless microscopy. The fundamental principle of our mathematical model is based on the phase-masking scheme^16^. This model is further coupled with the multi-angle illumination scheme to obtain the high-resolution image. The plane-wave relation in the x-y plane is given in Eq. (1).

1 $$W(x,y)=\exp (i{{\phi }}_{w}(x,y))=\exp (ik.\sin (m\pi x+n\pi y)).$$

The complex optical field behind the phase mask is given in Eq. (2).

2 $$\begin{array}{rcl}t(x,y) & = & W(x,y)P(x,y)\\  & = & \exp (i{k}_{z}.\sin (m\pi x+n\pi y)).\exp (i{{\phi }}_{p}(x,y))\\ t(x,y) & = & \exp (i.{{\phi }}_{r})\end{array}.$$

In the above equations, mπ and nπ are incident angles. ${{\phi }}_{r}={{\phi }}_{w}+{{\phi }}_{p}$ is the phase distribution after the phase mask, while ${{\phi }}_{p}\in [0,\,2\pi ]$ and *φ*_*w*_ show the phases of the phase mask and the plane waves, respectively. Throughout this article, the coordinates for the phase-mask plane, sample plane, and sensor plane are represented by (*x*,*y*), $({x}^{/},{y}^{/})$, and $({x}^{//},{y}^{//})$, respectively. In Eq. (1), the “m” and “n” causes a shift to the illumination beam with respect to the optical axis along both the x-axis and y-axis.

$O({x}^{/},{y}^{/})$ shows the tested sample, which has a transparent part, and is represented by the first term of Eq. (3).

3 $$O({x}^{/},{y}^{/})=1+a\,\exp (i{{\phi }}_{0}).$$

In Eq. (3), “a” is the amplitude and “φ_0_” is the phase of the sample. The plane-wave field is propagated from the source to the sample plane, which covers a distance of *z*_1_ along the optical axis. The field at the sample plane is given by Eq. (4)

4 $$S({x}^{/},{y}^{/})={P}_{P\to s}[t(x,y)].(1+a\,\exp (i{{\phi }}_{0}))$$

The optical field at three different planes is represented by Eq. (5). The subscripts “p,” “s,” and “sr” represent the phase mask, sample, and sensor plane, respectively.

5 $$\begin{array}{c}{P}_{p\to s}(t(x,y))={t}_{s}({x}^{/},{y}^{/})\\ {P}_{s\to sr}({t}_{s}({x}^{/},{y}^{/}))={t}_{sr}({x}^{//},{y}^{//})\end{array}$$

The sample field is propagated to the sensor plane at a distance of *z*_2_, where a hologram is directly recorded as a result of the interference of the diffracted object field and the undiffracted field, using a CMOS sensor, as given in Eq. (6). The propagation function is represented by “P,” while the propagating planes are represented by subscripts throughout the article.

6 $$I({x}^{//},{y}^{//})=\,{P}_{s\to sr}[S({x}^{/},{y}^{/})]={[{t}_{sr}({x}^{//},{y}^{//})+a.{t}_{sr}({x}^{//},{y}^{//})\exp (i{{\phi }}_{0}({x}^{//},{y}^{//}))]}^{2}.$$

The first term inside the bracket represents the undiffracted beam, while the second term represents the field after interacting with the elements of the sample. This co-axis interferogram in Eq. (6) is interesting because it allows for a stable and compact system, while addressing the twin-image issue at the sensor plane. Equation (6) is expanded to obtain two DC terms and two complex terms, where one is a real image and needs to be propagated back to the sample plane, and the second one is a twin image that needs to be excluded from the final image.

7 $$\begin{array}{rcl}I({x}^{//},{y}^{//}) & = & {|{t}_{sr}({x}^{//},{y}^{//})|}^{2}+{|a.{t}_{sr}({x}^{//},{y}^{//})\exp (i{{\phi }}_{0}({x}^{//},{y}^{//}))|}^{2}\\  &  & +(a.{t}_{sr}({x}^{//},{y}^{//})\exp (i{{\phi }}_{0}({x}^{//},{y}^{//}))){({t}_{sr}({x}^{//},{y}^{//}))}^{\ast }\\  &  & +\,{t}_{sr}({x}^{//},{y}^{//}){(a.{t}_{sr}({x}^{//},{y}^{//})\exp (i{{\phi }}_{0}({x}^{//},{y}^{//})))}^{\ast }\end{array}$$

Initially, the optical system does not have any samples, and an image is recorded to calibrate the system. This image carries the phase-mask and plane-wave information, and is given in Eq. (8).

8 $${I}_{r}={|{t}_{sr}({x}^{//},{y}^{//})|}^{2}.$$

Equation (8) is subtracted from Eq. (7) to eliminate the amplitude information of the phase mask. Then, the resultant equation is further evaluated to obtain the phase of the sample. The amplitude of light scattered from the sample features is relatively small. Then, the square of the amplitude further decreases the relative contribution towards the overall intensity. Therefore, the self-interference term is ignored.

9 $$\begin{array}{c}a.{t}_{sr}({x}^{//},{y}^{//}).\exp (i.{{\phi }}_{0.}({x}^{//},{y}^{//}))=\frac{(I-{I}_{r})}{{({t}_{sr}({x}^{//},{y}^{//}).)}^{\ast }}\\ \quad \quad \quad \quad \quad \quad \quad \quad \quad \quad \quad \quad \quad \,\,\,-\frac{({(a.{t}_{sr}({x}^{//},{y}^{//}).\exp (i.{{\phi }}_{0.}({x}^{//},{y}^{//})))}^{\ast }{t}_{sr}({x}^{//},{y}^{//}))}{{({t}_{sr}({x}^{//},{y}^{//}).)}^{\ast }},\end{array}$$

10 $$\begin{array}{c}{P}_{sr\to s}[a.\exp (i.{{\phi }}_{0.}({x}^{//},{y}^{//}))]={P}_{sr\to s}[\frac{(I-{I}_{r})}{{({t}_{sr}({x}^{//},{y}^{//}).)}^{\ast }}]/{P}_{sr\to s}({t}_{sr}({x}^{//},{y}^{//}))\\ \quad \quad \quad \quad \quad \quad \quad \quad \quad \quad \quad \quad -\frac{{P}_{sr\to s}([{(a.{t}_{sr}({x}^{//},{y}^{//}).\exp (i.{{\phi }}_{0.}({x}^{//},{y}^{//})))}^{\ast }{t}_{sr}({x}^{//},{y}^{//}).]/{({t}_{sr}({x}^{//},{y}^{//}))}^{\ast })}{{P}_{sr\to s}({t}_{sr}({x}^{//},{y}^{//}))},\end{array}$$

11 $$\begin{array}{c}a.\exp (i.{{\phi }}_{0.}({x}^{/},{y}^{/}))=\frac{{P}_{sr\to s}[(I-\,{I}_{r}).\exp (i.{{\phi }}_{r}({x}^{//},{y}^{//}))]}{{t}_{s}({x}^{/},{y}^{/})}\\ \quad \quad \quad \quad \quad \quad \quad \quad \quad -\frac{{P}_{sr\to s}([{(a.t({x}^{//},{y}^{//}).\exp (i.{{\phi }}_{0.}({x}^{//},{y}^{//})))}^{\ast }t({x}^{//},{y}^{//}).]/{(t({x}^{//},{y}^{//}))}^{\ast })}{{t}_{s}({x}^{/},{y}^{/})},\end{array}$$

12 $$a.\exp (i.{{\phi }}_{0.}({x}^{/},{y}^{/}))=\frac{{P}_{sr\to s}[(I-\,{I}_{r}).\exp (i.{{\phi }}_{r}({x}^{//},{y}^{//}))]}{{t}_{s}({x}^{/},{y}^{/})}-\,speckle\,noise,$$

13 $$speckle\,noise=\exp (i(-2{{\phi }}_{r}({x}^{/},{y}^{/})-{{\phi }}_{0.})({x}^{/},{y}^{/})).$$

This simple phase retrieval is for the normal illumination of coherent plane waves. To improve the resolution of the imaging system, a multi-angle illumination scheme was implemented. The technique is divided into two steps: imaging without a sample and imaging with a sample. The plane-wave equation is modified according to the range [−*θ*,*θ*] along the x-axis and y-axis symmetrically. For multi-angle illumination, the plane-wave equation (1) is modified as:

14 $${W}_{m,n}(x,y)=\sum _{-(m,n)}^{m,n}\exp (ik\,\sin (m\pi x+n\pi y)).$$

For a normal illumination angle, (*m*,*n*) corresponds to zero values. The other values of (*m*,*n*) produce a tilt in the illumination beam in the x-y plane. This tilt basically shifts the spectrum of the sample at the sensor plane. When illuminating the sample along a single direction, the value of “m” varies, while “n” is constant, and vice versa for other perpendicular directions. Both types of images (with the sample and without the sample) are captured for each tilted illumination beam. To eliminate the phase of the tilted beam and phase mask, the images with the sample are subtracted from the images without the sample for each tilt. To eliminate the effect of the phase mask in the resultant image, the complex field without the sample is computed and divided from the subtraction result mentioned above. This process is repeated for all images, and the resultant images are summed at the sample plane. The resultant image is propagated to the sample plane, which has an enhanced resolution when compared to the image captured with normal illumination. The multi-angle phase and amplitude retrieval, which limits the equation to producing a single image with significant resolution, is given below.

15 $$a.\exp (i.{{\phi }}_{0.}({x}^{/},{y}^{/}))=\sum _{-(m,n)}^{m,n}\frac{{P}_{sr\to s}(({I}_{m,n}-{I}_{r,m,n}).\exp (i.{{\phi }}_{r,m,n}({x}^{//},{y}^{//})))}{{t}_{s,m,n}({x}^{/},{y}^{/})}+speckles\,noise.$$

To reduce this equation for conventional imaging, consider the case where only normal illumination is implemented and two images (with sample and without sample) are recorded with (*m* = 0, *n* = 0) in Eq. (15). Equation (15) is reduced to Eq. (12) with low resolution and a weak SNR.

$$a.\exp (i.{{\phi }}_{0.}({x}^{/},{y}^{/}))=\frac{{P}_{sr\to s}(({I}_{0,0}-{I}_{r,0,0}).\exp (i.{{\phi }}_{r,0,0}({x}^{//},{y}^{//})))}{{t}_{s,0,0}({x}^{/},{y}^{/})}+speckles\,noise.$$

The speckle noise in the background of the resultant image is equally distributed across the image. The sample features only occupy a small proportional FOV, and the even distribution of the noise (twin-image term) effect throughout the sample does not degrade the image quality severely.

## Methods

**Figure 1:**
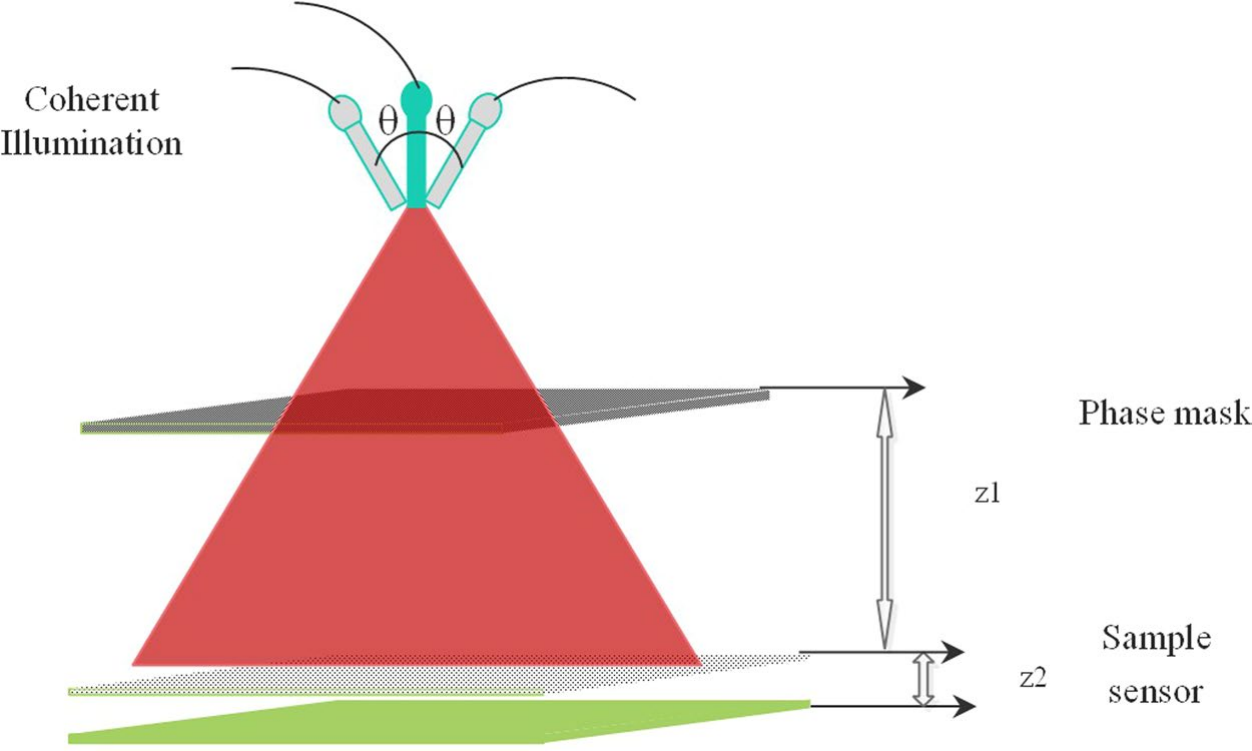
Optical system for multiple angles of illumination; Illumination beam has angles from −ve to +ve with symmetrical geometry, while z1 and z2 are the phase mask of the sample and sample-to-sensor distance, respectively.

The experimental setup to validate the concept of lensless microscopy using the phase-masking technique is given in Fig. 1. The three main components of the experimental setup are the illumination beam, phase mask, and sensor. In Fig. 1, a coherent source of wavelength 641 nm from Coherent Company is coupled through the optical fiber to ensure spatial coherence. The phase mask with random phase values ranging from 0 to 2π is generated using MATLAB and assigned to a spatial light modulator (SLM) (Daheng Company). The pixel size of the SLM is 12 μm, and the SLM operates in the transmission mode. The ideal pixel size of the SLM is ~1 μm, which is comparable to that of the sensor. The sample is USAF 1951 resolution chart (Stock No. #58198) obtained from Edmund optics, and is placed at a distance of z_1_ from the phase mask along the optical axis, and at a distance of z_2_ from the sensor. The value of z_1_ is of the order of a few centimeters, i.e., (9~10 cm) while the distance z_2_ is 800~1000 μm for our sensor. A CMOS sensor with a pixel size of 1.4 μm and dimensions 3264 × 2448 pixels was used to record the holograms of the transmission-mode sample. The CMOS used in this experiment was manufactured by Sony Company. Because of the electronic circuit geometry, z_2_ is limited to the above-mentioned value. This restriction also directly affects our resolution as z_2_ cannot be decreased further. The procedure for multi-angle illumination is very straightforward in that the rotation arm is used to produce a tilted illumination. However, the accuracy of this rotation arm is not ideal as it also produces some shifts between the sample image and reference image for the same angle. This shift is corrected during the post processing of the images. This small shift also decreases the SNR as the background is not completely canceled during the subtraction process. The multi-angle illumination is a systematic process in which the rotation arm, having the source and phase mask relatively fixed, is shifted using the program LabVIEW, to the maximum angle of −30° with the optical axis. To calibrate the system, we captured a total of 31 images with an angular step size of 2°. In the next step, the sample is placed at distance z_2_ from the sensor and 31 images are recorded by repeating the same procedure. Owing to these multiple angles of illumination, the images at the sensor are shifted corresponding to the changing beam direction, and they are stored in the computer memory. All of the 62 images are back shifted to a central position with respect to the normal illumination angle using the computation process^18,19^. The two types of images are then further processed following the model given in the previous section to obtain the final image. To scan the y-axis of the system, the sample is rotated in-plane by an angle of 90° with respect to the system axes. Owing to the rectangular shape of the sensor geometry, this rotation also limits the FOV. To avoid this situation, the sensor and sample can be relatively fixed and rotated through an angle of 90°. For this direction, a total of 31 images are recorded and subtracted from the already recorded reference images to eliminate the background noise. The rotation of the sample and sensor is easy compared to the source rotation with an angle of 90°. The value of z_2_ is important, but somewhat complex to measure. z_2_ is determined computationally, where the image sharpness is measured. To obtain a more precise measurement, the step size for z_2_ within the range is kept small, and the image sharpness determines the final z_2_ value. Further, to more precisely control the z_2_ value, the sample dimensions should be sufficiently small to avoid any hurdle from circuit elements.

In this study, the SLM is used primarily because of its in-house availability. A passive phase mask can be used instead of an SLM to carry out the experiment as long as the passive phase mask has a similar or higher resolution (i.e., high spatial frequencies in the phase distribution). Its use as a passive phase mask will also make the system portable.

**Figure 2:**
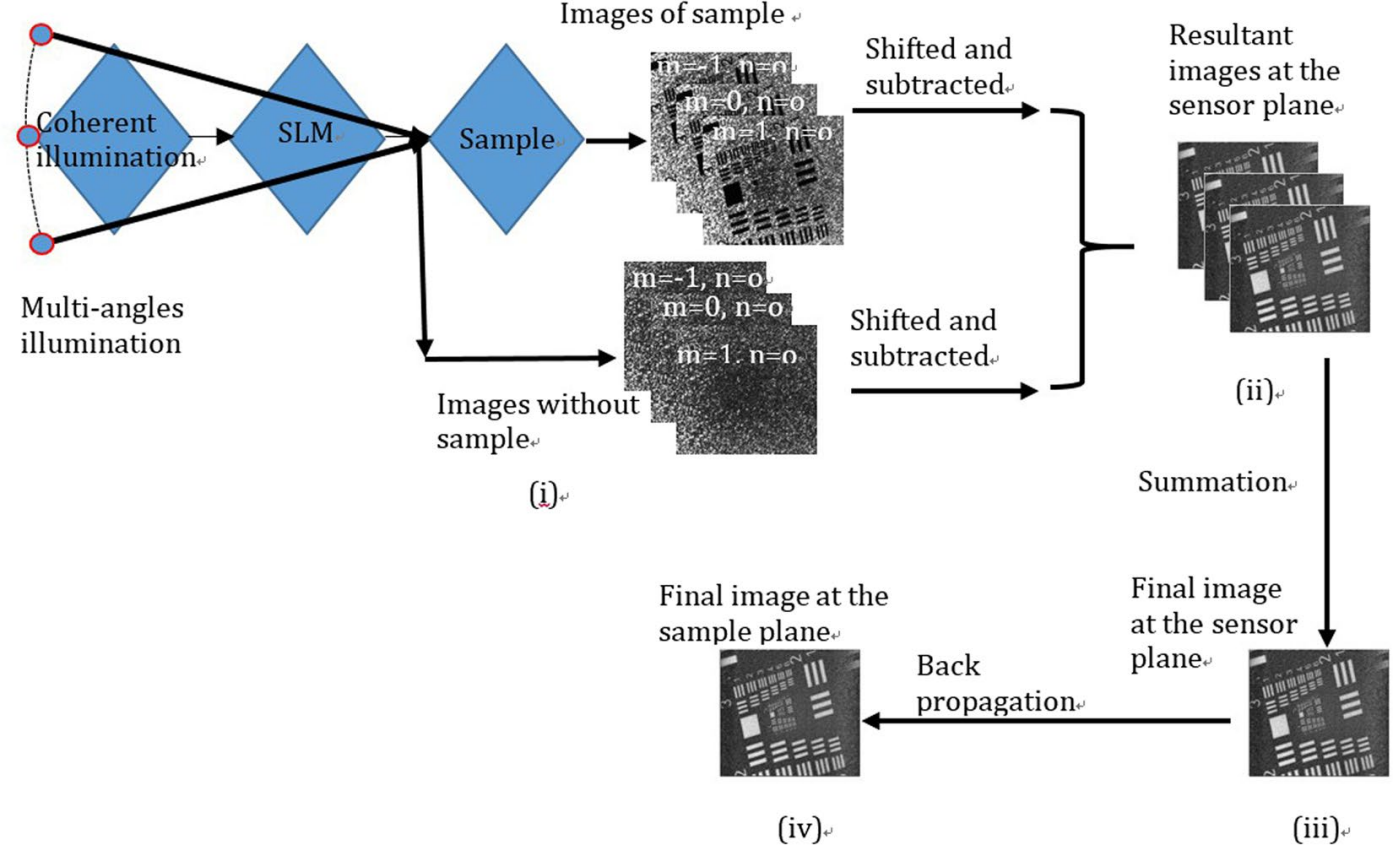
Flow chart: The four-steps of the image registration and reconstruction process are explained in the pictorial view. (**i**) Image registration with sample and without sample. (**ii**) Recorded images are shifted and subtracted. (**iii**) Resultant images are added. (**iv**) The summed image is backpropagated through the angular spectrum method to the sample.

The whole process of lensless microscopy using a phase mask is summarized in the schematic diagram shown in Fig. 2. Figure 2(i–iv) illustrates the capturing of images both with and without the sample, after which they are shifted to the center and subtracted, summed, and back propagated to their respective sample planes. For simplicity, the step at which the subtraction result is divided by the reference complex field is omitted from this flowchart.

### Shifting procedure

The spectrum recorded at the sensor plane is a shifted version of the original sample spectrum owing to the multi-angle illumination scheme. All of the recorded images, both with sample and without the sample, have different shifts that correspond to their imaging angles. The two types of images are back shifted to the center using an image-registration technique^18,19^, based on similarity between elements. In this technique, a single element of the sample image is selected as a reference for comparison in all images, and these images are shifted to the position of the central images on the basis of this selection. The reference images are also shifted using the same shifting parameters with respect to the central sample image. To further improve the accuracy of the back-shifting of images up to a few pixels, an optimization procedure is applied, where the subtracted images are converted to binary images (i.e., black and white). Then, a series of fine-shifting values are tested to find the value that gives the largest overlap ratio among the white features in the binary images, and which results from different angles of illumination. When the overlap ratio reaches a maximum, the images are back-shifted to exactly the same position, which means that the shifting caused by multi-angle illumination is completely corrected. Note that this back-shifting procedure is entirely computational and automatic, without the need for any artificial adjustments.

### Data availability statement

The datasets generated and/or analyzed during the current study are available from the corresponding author upon receiving a reasonable request.

## Results and Discussion

The validity of this technique was verified both theoretically and experimentally. In lensless microscopy, the sample elements scatter coherent light, and the empty space of the sample gives unscattered light, which acts as the reference beam. The interference of the two beams at the sensor produces the hologram, which is shifted in a manner corresponding to the tilted beam. The angle of each tilted beam is measured with respect to the normal beam. All of the recorded holograms are back-shifted to their proper position using computational algorithms^18,19^. The FOV of the final image is limited by the dimensions of the sensor. The technique was tested for two dimensions using simulations as well as experimentally. In our case, the USAF 1951 resolution chart was used to test the phase-masking scheme. The resolution of our system with defined parameters is 4 μm.

**Figure 3:**
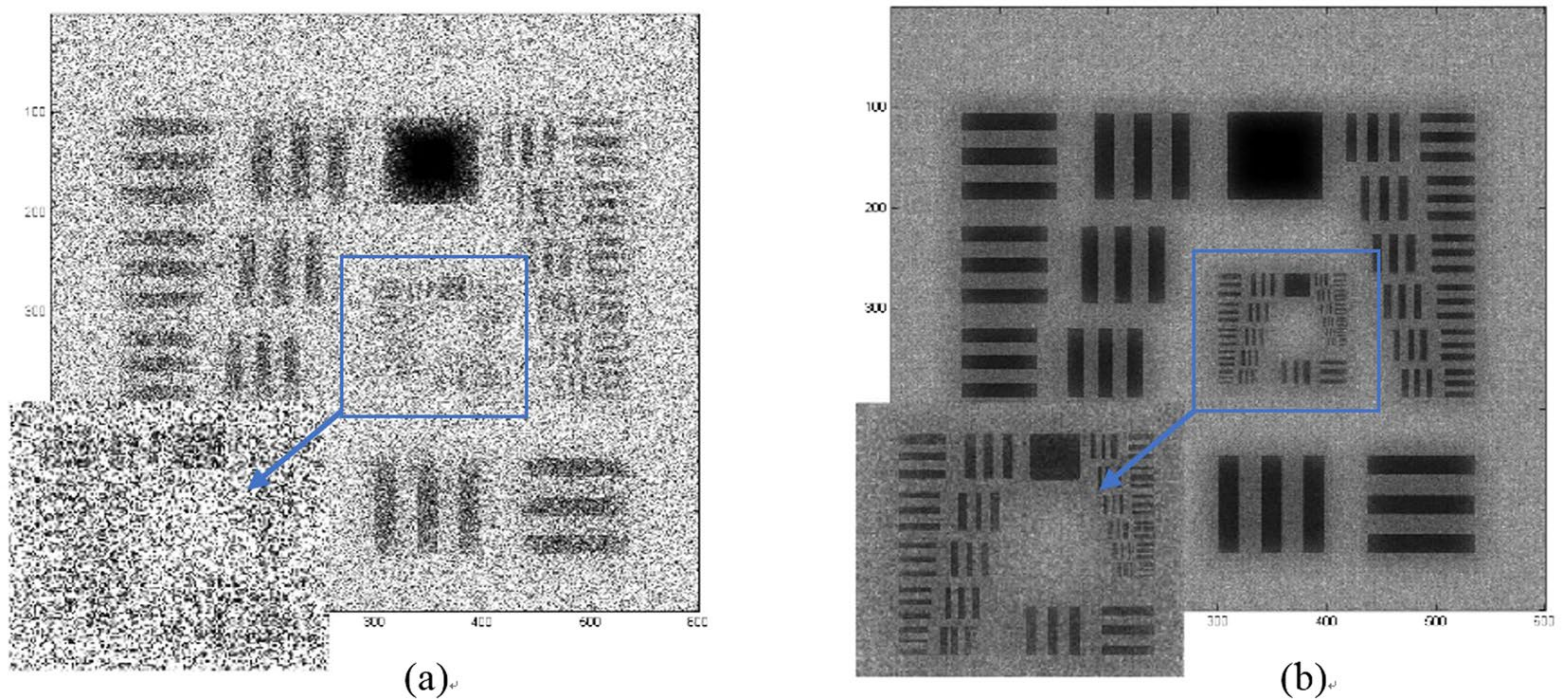
Comparison of simulation results. (**a**) Conventional image obtained with single normal illumination angle (**b**) Super-resolved image obtained using multi-angle illumination. The central section of both images is magnified for a detailed comparison.

**Figure 4:**
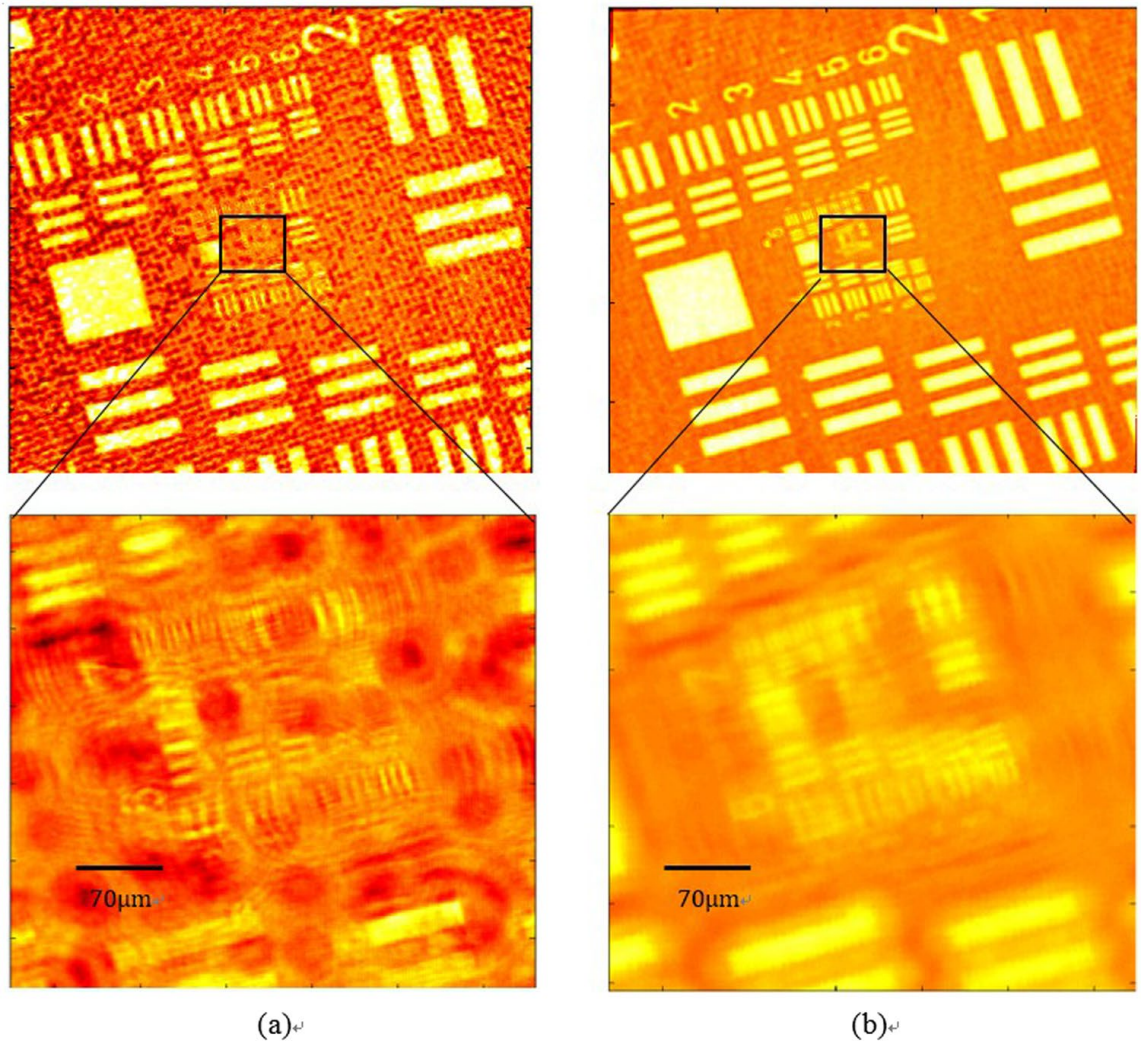
Experimental results; comparison of normal illumination angle image with super-resolved image. (**a**) Image obtained from normal illumination with a magnified central section (**b**) image obtained from multi-angle illumination with a magnified central section.

**Figure 5:**
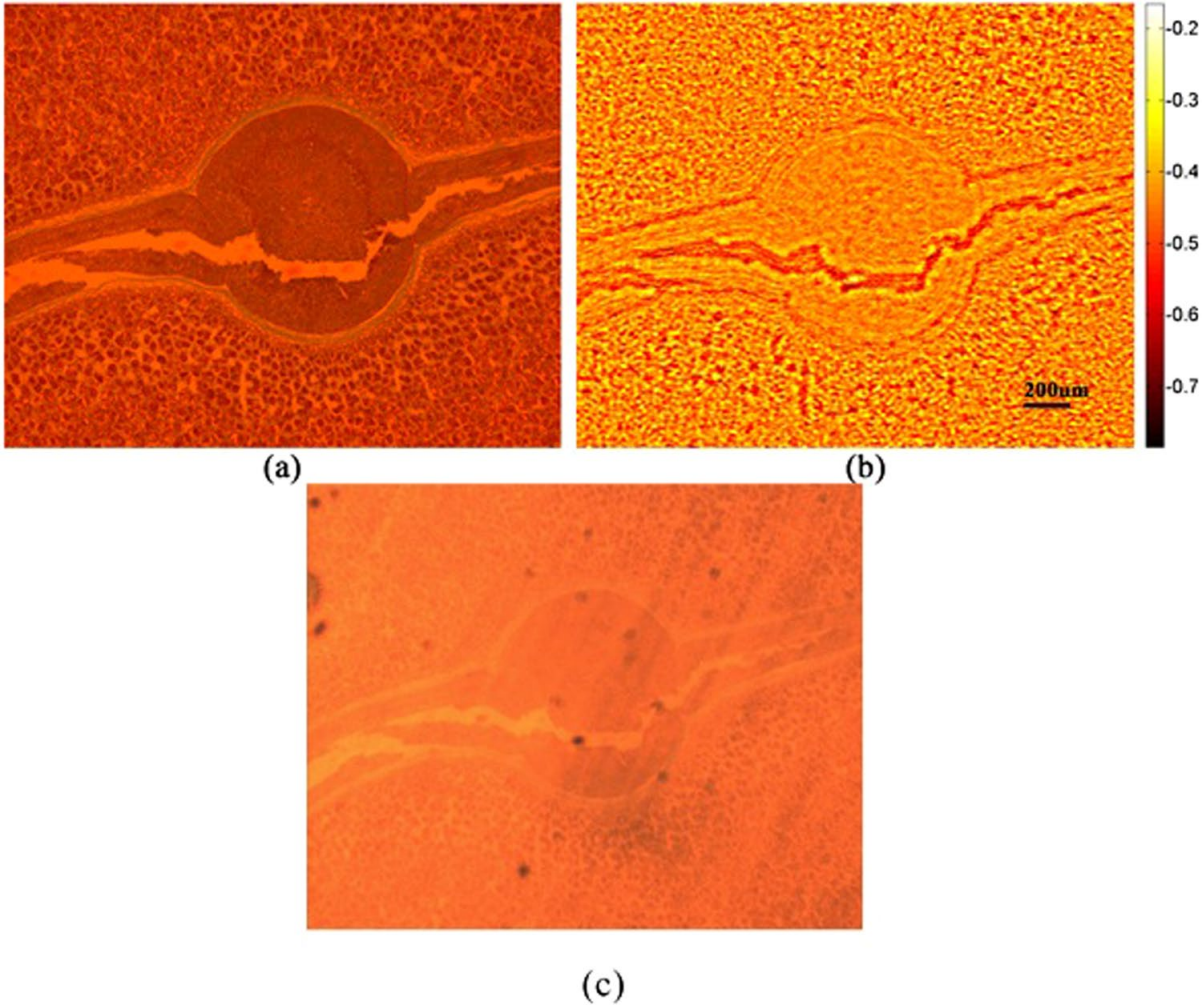
Phase image of a seed of castor plant using (**a**) phase-contrast microscopy and (**b**) the proposed method. Color bar shows the retrieved phase values in radians. (**c**) bright field in focus image.

Figure 3 shows the simulation results of the normal illumination image and the super-resolved image for comparison purposes. The central portion in both images is cropped and magnified to aid visual comparison. The maximum resolution of Fig. 3(b) is 4 μm, which is higher compared to Fig. 3(a). The image with a normal illumination, which is called a conventional image, has a low SNR, and the sample elements’ information is not cleared compared to Fig. 3(b). The comparison of the two images clearly indicates the removal of the twin image. The noise is not completely removed from the final image owing to the twin-image distribution throughout the image dimensions. This noise factor does not affect the resolution, but it affects the contrast of the image to some degree. The simulation conclusions are further strengthened by performing experiments, and the results are presented in Fig. 4. The USAF 1951 resolution chart from Edmund Optics (Stock No. #58198) has a resolution of ~4 μm for group 7. In our experimentation, group 7 is resolved, as shown in Fig. 4. The comparison in Fig. 4 shows the improvement in the resolution for the group of elements, which were not resolved for the conventional image. The image given in Fig. 4(b) was obtained from 93 images. The multi-angle illumination technique therefore improves the SNR as well as the resolution. To compare the resolution values, the central part is selected and observed. Both the vertical and horizontal lines are resolved for groups 6 and 7. The only difference between the simulation and experimental parameters is the distance z_2_, which is limited during the experiments. The sensor from Sony Company was not manufactured for lensless microscopy applications, and the specifications of the electronic elements on the sensor chip determine the minimum possible sample-to-sensor distance. To further improve the resolution, this technique can be coupled using source or sensor shifting at the sub-micron level to beat the pixel-resolution limit. The proposed scheme was further tested for the phase retrieval of a seed of castor plant. The main purpose of this additional work is to verify the scheme that is employed for phase retrieval. The phase images are given in Fig. 5. While Fig. 5(a) shows the phase image obtained using phase-contrast microscopy, the phase image retrieved with the proposed method using five raw images is shown in Fig. 5(b). Figure 5(c) shows the bright field image of the phase sample in focus. The microscope used for the phase-contrast image and bright field image is a Nikon LU Plan Flour with a numerical aperture of 0.15 for a × 5 magnification. The phase of the main features is correctly reconstructed. The peripheral parts appear to be slightly different from the phase-contrast image, and this is mainly because the phase features in these regions are denser, which makes it more difficult to realizeaccurate reconstruction.

**Figure 6:**
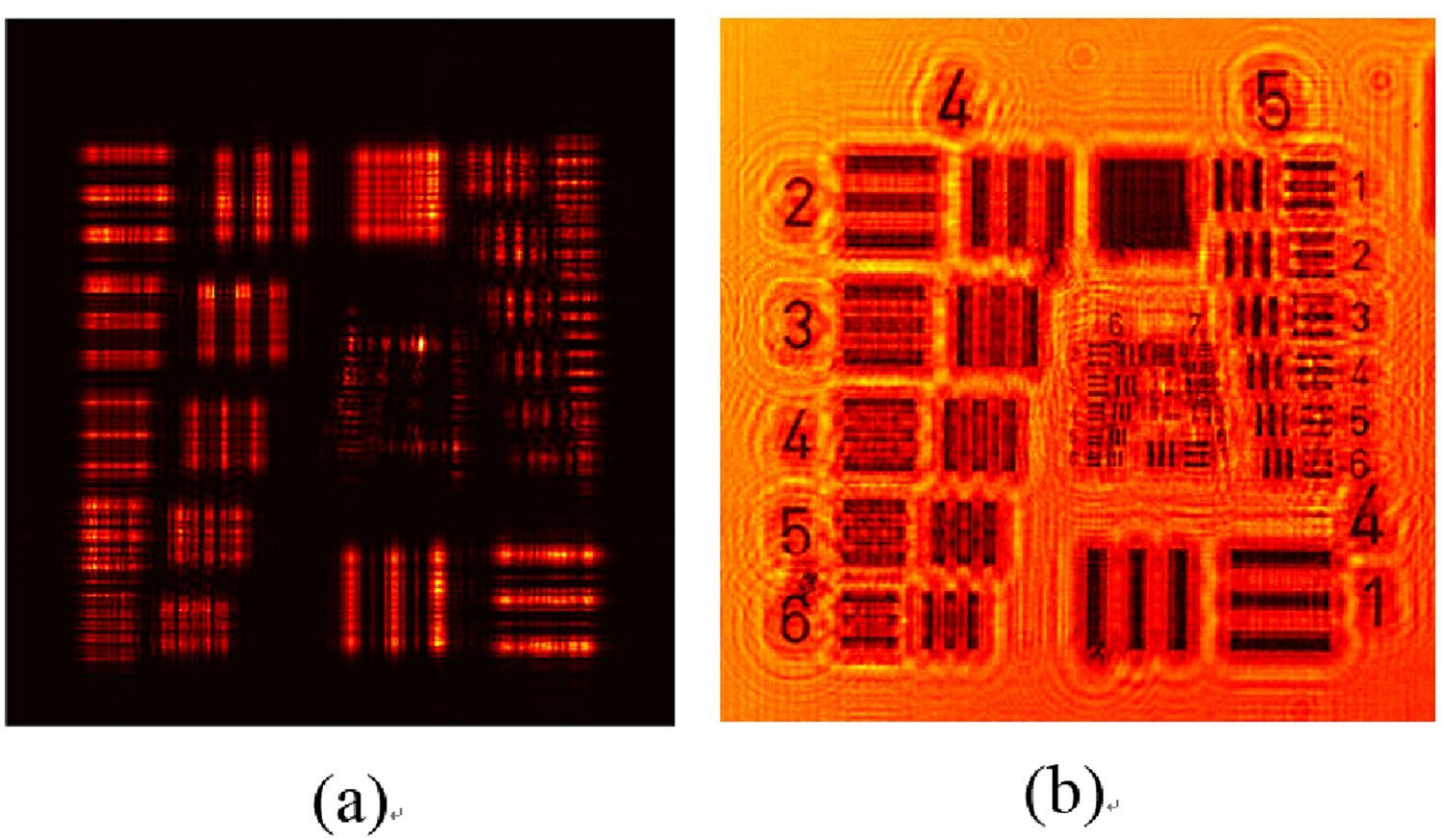
Images without phase mask: (**a**) Simulation image. (**b**) Experimentally retrieved image. The twin-image artifacts are apparent.

**Figure 7:**
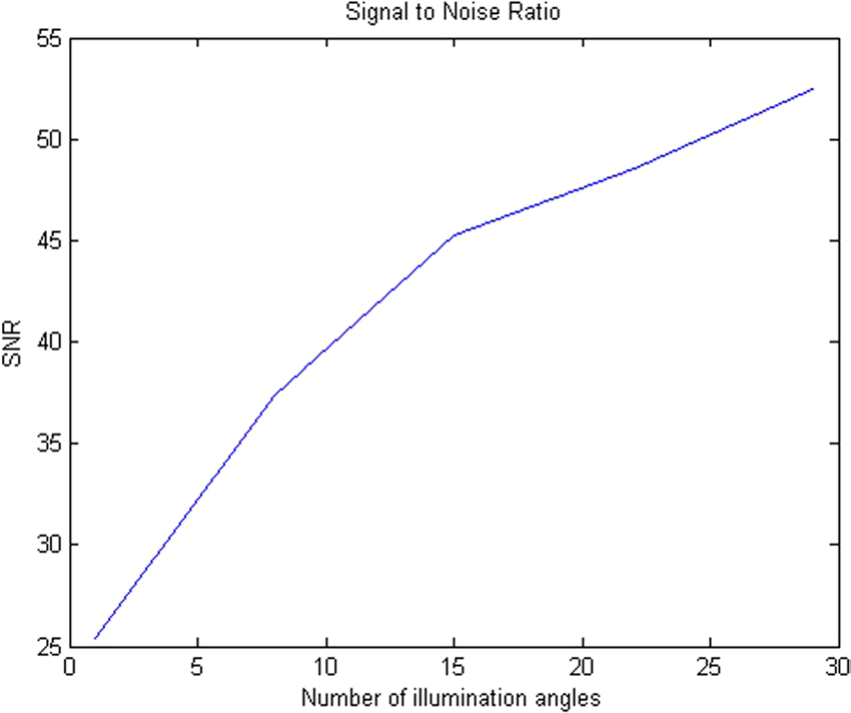
Signal-to-noise ratio (SNR) versus number of illumination angles.

Figure 6 shows the single-illumination images obtained from the simulation and experimentation using our parameters without the phase mask. It can be observed that severe twin-image artifacts appear when no phase mask is used. In fact, the initial reason for introducing the phase mask is to suppress the twin image. Figure 7 shows the SNR versus the number of illumination angles. The SNR increases as the number of illumination angles increases. The graph is observed to be saturated for higher values of angles, which implies that a further increase in the illumination angles does not increase the amount of sample information collected, but only increases the background noise. For the USAF 1951 resolution chart, the misalignment of the sample axis and the axis of illumination also affects the SNR.

The total time required in the proposed method is divided into two blocks: the time to capture raw images, including calibration images, and the computation time. It should be noted that the calibration images are all recorded once, and this operation does not need to be repeated for every sample. This reduces the number of raw images and increases the speed of the method. The time required to capture images is limited by the speed of the rotation arm controlled by LabVIEW, and is approximately 2 s per image. In the case of our experiment shown in Fig. 4, where 62 images with objects and 31 calibration images are used, the capture process takes approximately 3 min. The raw images are post-processed using MATLAB, for which the speed depends on the PC specifications. In this study, an ordinary laptop was used, and it takes around 20 min to process 93 images, each having 3264 × 2448 pixels. The algorithm has almost no iterations, which significantly reduces the computation time. The scheme presented here is an advanced configuration of the scheme presented in ref.^16^, where the random-phase mask was used to retrieve the sample information up to the 63-μm resolution limit. In our scheme, the multi-angle illumination diversity is utilized to capture the high spatial frequencies of the sample to be retrieved ~4 μm, which achieves a × 16 improvement in the resolution. A close comparison shows that the proposed scheme is significantly improved in terms of resolution, compactness of the system, and relative data efficiency than [17]. Compared with ref.^11^, in the proposed scheme, 31 images were recorded with the sample for ~4-μm resolution along one direction, while the reference images (without the sample) were recorded once during the entire process. In [11], 44 images were recorded with a resolution <1 μm, and the smaller number of images improved the data efficiency. There are two differences between the proposed scheme and ref.^11^. First, in [11], the sample-to-sensor separation is ~200 μm, while in this study, it is ~780 μm. The resolution of the proposed scheme can be further improved if this distance is reduced. Second, the reconstruction process is different. The method in [11] incorporated the pixel super-resolution technique, while in this study, only noniterative reconstruction was used. The resolution of the proposed scheme is less than the size of the sensor pixel, not because of the usage of the phase mask but owing to the above-mentioned limiting experimental parameters. A portable version of the system can be easily built if the SLM is replaced with a passive phase mask and the rotation arm is replaced with an array of illumination sources. Possible sources include a bunch of fiber-coupled laser sources, which are turned on sequentially to produce multi-angle illumination.

## Conclusions

The lensless phase-masking scheme is a novel reconstruction method that can be used to retrieve sample information. This new scheme not only improves the resolution and FOV but also simplifies the reconstruction process. The image reconstruction process is simplified without altering the recording process. This scheme is a compact, cost effective, and portable system, and has a high transverse resolution and large FOV as additional advantages. The geometry of the CMOS sensor and rotation arm can be modified to obtain high resolution with the same setup. To compensate for the pixel size of the sensor, this technique can be combined with the x-y scanning of a sensor that has a subpixel size. The phase retrieval is based on the phase mask, which will be used in future to enable 3D imaging.
